# Awake versus deep airway device removal in pediatric tonsillectomy: a systematic review and meta-analysis

**DOI:** 10.1016/j.bjane.2026.844840

**Published:** 2026-10-09

**Authors:** Henrique Baggio Pivetta, Alice O. Almeida, Rafael L. Miranda, Rafaela P. Silva, Raphaela G. Mendes, João M.M. Borges, Raquel E. Saldanha, Matheus Arraes, Pablo P. Sanín, Walid Alrayashi

**Affiliations:** a Universidade do Sul de Santa Catarina, Department of Medicine, Palhoça, SC, Brazil; b Faculdade de Medicina Zarns, Department of Medicine, Salvador, BA, Brazil; c Universidade Federal de Minas Gerais, Department of Medicine, Belo Horizonte, MG, Brazil; d Universidade Federal do Maranhão, Department of Medicine, São Luís, MA, Brazil; e The University of Texas Health Science Center at Houston, Houston, TX, USA; f Hospital Instituto José Frota, Anesthesiology Department, Fortaleza, CE, Brazil; g University of Miami Miller School of Medicine, Miami, FL, USA; h Colsubsidio Cardiovascular Clinic, Bogotá, Colombia; i Boston Children's Hospital and Harvard Medical School, Anesthesiology Department, Critical Care, and Pain Medicine, Boston, Massachusetts, USA

**Keywords:** Adenoidectomy, Child, Extubation, Intubation, intratracheal, Laryngeal masks, Tonsillectomy

## Abstract

**Background and objectives:**

Tonsillectomy, with or without adenoidectomy, is among the most common pediatric surgeries, associated with a high incidence of perioperative respiratory adverse events. The timing of airway device removal, during deep anesthesia or after awakening, may influence these outcomes.

**Methods:**

PubMed, Embase and CENTRAL were systematically searched for studies comparing awake and deep airway device removal in pediatric otolaryngology surgery. The primary analysis was restricted to Randomized Controlled Trials (RCTs). The primary outcome was laryngospasm; secondary outcomes included cough, oxygen desaturation and airway obstruction. Risk Ratios (RR) with 95% Confidence Intervals (95% CI) were pooled using random-effects models.

**Results:**

Seven studies (six RCTs and one observational study, the latter used only in sensitivity analyses), including 1,571 children, were analyzed. Deep airway device removal was associated with a higher risk of airway obstruction (RR = 1.82; 95% CI 1.10–3.03; p = 0.020) and a lower risk of cough (RR = 0.57; 95% CI 0.43–0.75; p < 0.001), with no evidence of a difference between the Laryngeal Mask Airway (LMA) and Endotracheal Tube (ETT) subgroups (p for subgroup differences = 0.992). Laryngospasm did not differ significantly (RR = 0.99; 95% CI 0.43–2.30; p = 0.984). Desaturation was lower with deep removal in the RCT-only analysis (RR = 0.62; 95% CI 0.44–0.88; p = 0.007).

**Conclusions:**

Deep airway device removal was associated with less coughing, with no evidence that this effect differed between LMA and ETT. Deep removal may increase airway obstruction, though sensitive to individual studies. Desaturation findings were sensitive to study design and are not definitive.

## Introduction

Adenotonsillectomy and tonsillectomy are among the most frequently performed surgical procedures in the pediatric population, primarily indicated for the treatment of upper airway obstruction and recurrent infections of the tonsils and adenoids.^1^, ^2^, ^3^, ^4^, ^5^, ^6^ At the end of the procedure, the removal of airway devices, including LMA and ETT, can be performed either while the patient remains in a deep plane of anesthesia, when spontaneous ventilation is established but airway reflexes have not yet returned, or after full emergence and awakening.^7^^,^^8^ Both techniques have critical respiratory safety implications, particularly in children, who are more susceptible to laryngospasm, coughing, desaturation, airway obstruction, and stridor due to heightened airway reactivity.^9^^,^^10^ These risks are further amplified during surgeries involving the upper airway, such as adenotonsillectomy or other oropharyngeal procedures, in which the presence of blood and secretions act as potent airway irritants.^9^^,^^11^^,^^12^ These complications highlight the importance of careful anesthetic management during adenotonsillectomy and tonsillectomy procedures in children.^6^

The transition to Total Intravenous Anesthesia (TIVA) and sevoflurane largely replaced older volatile agents like halothane and enflurane. This shift prioritized superior safety, faster and smoother recoveries, and lower toxicity, addressing specific clinical challenges associated with the older medications. Understanding whether modern anesthetic practice mitigates or exacerbates the classic risks associated with each extubation technique is essential for evidence-based perioperative management, particularly in high-risk patients.

However, evidence comparing the available methods of airway device removal (deep vs awake) in tonsillectomy and adenotonsillectomy patients remains conflicting.^8^^,^^13^^,^^14^ In contrast to a 2018 meta-analysis, which evaluated airway device removal timing in a general pediatric population without surgical stratification, the present study focuses specifically on otolaryngology procedures and incorporates recent evidence to provide an updated synthesis.^8^ This meta-analysis aims to clarify whether removal of the LMA or ETT under deep anesthesia or after full emergence results in superior respiratory safety and recovery outcomes in pediatric patients.

Importantly, much of the foundational literature addressing extubation depth in pediatric tonsillectomy was conducted during earlier anesthetic eras, prior to widespread use of sevoflurane and TIVA. As a result, existing data may offer mechanistic insight rather than direct guidance for contemporary practice.

## Methods

This systematic review and meta-analysis was pre-registered on the International Prospective Register of Systematic Reviews (PROSPERO) with protocol number CRD420251084368. Institutional ethics approval was waived. Data registration, conduct of the meta-analysis, and reporting adhered to the guidelines outlined in the Cochrane Handbook for Systematic Reviews of Interventions and the Preferred Reporting Items for Systematic Reviews and Meta-Analyses (PRISMA) statement.^15^^,^^16^ The complete PRISMA checklist is available in Supplementary Methods 1.^17^

### Search strategy

A comprehensive search was conducted in MEDLINE, Embase, and the Cochrane Central Register of Controlled Trials (CENTRAL) from inception to September 2026. The complete search strategy is provided in Supplementary Methods 2. Two reviewers independently screened studies for eligibility and extracted data using a predefined extraction form. Discrepancies were resolved by consensus.

### Eligibility criteria

Studies were included if they met all of the following: 1) RCTs were eligible for the primary meta-analysis; 2) Comparing awake and deep removal of airway devices; 3) Conducted in pediatric patients undergoing tonsillectomy and adenotonsillectomy; and 4) Reporting at least one predefined outcome. Observational studies meeting criteria (2) through (4) were considered separately and included only in sensitivity analyses.

Articles were excluded if they (1) lacked a control group; (2) were conference abstracts; or (3) involved overlapping study populations. No date restrictions were applied.

### Outcome assessments

The primary outcome was laryngospasm. Secondary outcomes included cough, oxygen desaturation, and airway obstruction. Laryngospasm was defined as complete upper airway obstruction with absence of airflow, associated with inspiratory stridor and paradoxical chest wall movement. Cough was considered when persistent or severe (> 10s) coughing occurred during emergence or in the post-anesthesia care unit. Oxygen desaturation was recorded continuously by pulse oximetry, typically defined as SpO₂ below 92%‒95% for more than 30 seconds or requiring supplemental oxygen. Airway obstruction was identified clinically as partial obstruction accompanied by snoring, increased respiratory effort, or the need for airway maneuvers (chin lift or jaw thrust). Outcomes were assessed during the immediate postoperative period. Datawere pooled in meta-analyses when at least three studies reported a given outcome.

### Study selection and data extraction

Two reviewers (H.B.P. and A.O.A.) independently screened the studies in two phases: initial title and abstract screening, followed by full-text review. Discrepancies were resolved through discussion or consultation with a third reviewer (R.P.S.). Two authors (R.G.M. and J.M.M.B.) independently extracted the data for each study using a standardized study form to determine: authors, enrollment period, study publication year, inclusion and exclusion criteria, sample size, follow-up period, baseline patient characteristics, endpoint data and endpoint definition. Disagreements were resolved in a panel discussion with the first author (H.B.P.).

Additionally, all included studies and the references used were screened against the Retraction Watch Database (retractionwatch.com), PubMed/MEDLINE, publisher websites and CrossMark were consulted in September 2026. No retractions or expressions of concern were identified among the studies included.

### Statistical analysis

Risk Ratio (RR) was used for dichotomous outcomes, estimated via the Mantel-Haenszel (MH) method. Pooled estimates were calculated using random-effects models with REML estimators for τ² and 95% Confidence Intervals (95% CIs), considering potential variations in clinical and methodological factors across studies. Statistical significance was set at p < 0.05, with emphasis on effect size magnitude and precision. Analyses were conducted in RStudio (v4.5.0; RStudio, PBC) using the meta and metafor packages.

The primary meta-analysis was restricted to randomized controlled trials to minimize confounding and intervention-classification bias. The observational study was not included in the primary pooled estimates but was incorporated in a sensitivity analysis when outcome data were available. Additional exploratory subgroup analyses were performed according to airway device type and sevoflurane-based maintenance anesthesia.

Although ETT and LMA differ in their anatomical positioning, sealing mechanisms, and degree of airway stimulation, both devices share the same fundamental intervention under investigation, the timing of removal relative to depth of anesthesia. Furthermore, both devices are routinely used in the same surgical population (pediatric tonsillectomy), and the respiratory outcomes assessed are clinically relevant regardless of device type. To address the clinical heterogeneity introduced by device differences, subgroup analyses stratified by airway device type were performed for all outcomes, and results are interpreted in the context of these subgroups rather than the pooled estimate alone. The statistical significance of differences between subgroups was assessed with the test for subgroup differences (Cochran's *Q*), rather than by comparing the statistical significance of the individual subgroup estimates.

### Deviations from the registered protocol

Three minor deviations from the registered protocol are acknowledged. First, airway obstruction was added as a secondary outcome after protocol registration, based on its clinical relevance to the surgical population studied. Second, although the registered protocol specified inclusion of studies published in English, Spanish, Portuguese and Chinese, no language restrictions were applied during the search to maximize sensitivity and minimize publication bias; this decision was made prior to study selection. Third, the subgroup analyses by airway device type and anesthetic maintenance agent (sevoflurane) were not prespecified in the registration and should therefore be considered exploratory.

This subgroup included studies in which sevoflurane was used for anesthetic maintenance, regardless of induction agent or concomitant use of nitrous oxide. For most outcomes, only randomized controlled trials contributed to this subgroup analysis. For the desaturation outcome, Baijal et al. was the single observational study in this review, also included in the sevoflurane subgroup, as sevoflurane-based maintenance was used in that study.

### Publication bias assessment

Two independent reviewers assessed the risk of bias in the individual trials and the methodological quality of the included studies (H.B.P. and M.A.). Discrepancies were resolved by consensus. To evaluate the risk of bias in RCTs, we used the Cochrane Risk of Bias 2 (RoB2) tool (Suppl. Table 1).^18^ For each domain, we assigned a judgement of “high risk”, “some concerns” or “low risk” of bias. For the observational study, the Risk of Bias in Non-Randomized Studies of Interventions (ROBINS-I) tool was applied,^19^ assessing domains such as selection bias, performance bias, detection bias, attrition bias, and reporting bias (Suppl. Table 2).

The overall certainty of the evidence was evaluated using the Grading of Recommendations, Assessment, Development and Evaluation (GRADE) guidelines.^20^ Studies were rated as providing very low-, low-, moderate-, or high-quality evidence based on the presence of risk of bias, inconsistency of results, imprecision, publication bias, and the magnitude of treatment effects.

### Assessment of heterogeneity

Heterogeneity was assessed by visual inspection, the I² statistic, and Cochran’s *Q*-test. Given the small number of included studies, I² values should be interpreted with caution, as they may not reliably distinguish true homogeneity from insufficient statistical power to detect heterogeneity. Leave-one-out analyses were conducted with all studies. Sensitivity analyses incorporating the observational study were performed to explore the robustness of the findings and potential sources of heterogeneity.

## Results

### Study selection and characteristics

The initial literature review identified 739 records. After removal of duplicates and exclusion based on title and abstract screening, 13 articles were selected for full-text review. Of these, 3 were available only as conference abstracts and 3 were trial registries without published results. Consequently, 7 studies met the inclusion criteria and were included in the meta-analysis, six RCTs^10^^,^^11^^,^^21^, ^22^, ^23^, ^24^ contributed to the primary analysis and one observational study was used only in sensitivity analyses.^25^

A total of 1,571 patients with a mean age of 4.88 years (ranging from 1 to 19 years) underwent tonsillectomy or adenotonsillectomy, of whom 647 (41.2%) were extubated awake. The male proportion varied between 41% and 72%, and the weight range across studies spanned from approximately 12 kg to 26 kg. Four studies included patients undergoing adenotonsillectomy,^10^^,^^23^, ^24^, ^25^ whereas three studies included tonsillectomy with or without adenoidectomy.^11^^,^^21^^,^^22^ Five studies employed an ETT for airway management,^10^^,^^22^, ^23^, ^24^, ^25^ while two studies utilized LMA.^11^^,^^21^ Baseline physical status was generally low risk. Four studies included children classified as ASA I–II, and one study was limited to ASA I patients. One study enrolled patients with ASA physical status I–III. ASA classification was not reported in the observational study. Sevoflurane was the most commonly used induction and maintenance anesthetic agent, reported in five studies.^10^^,^^11^^,^^21^^,^^24^^,^^25^ One study^22^ employed halothane for both induction and maintenance of anesthesia while another^23^ used enflurane for both purposes. Four studies reported the presence of OSA,^10^^,^^11^^,^^24^^,^^25^ with a total of 385 children with obstructive sleep apnea in the awake extubation group and 264 who underwent deep extubation. Study characteristics are summarized in Table 1, and the PRISMA flow diagram is shown in Figure 1.

**Table 1:** Baseline characteristics of included studies.

Study, year	Study design	Surgery	ASA-PS	Airway device	Awake/ Deep, n	Age, y Awake/Deep^a^	Weight, kg Awake/Deep^a^	Male, n (%)	Induction	Maintenance	Diagnosis of OSA, n Awake/Deep
Patel, 1991	RCT	T/A	I‒II	ETT	18/20	4.04 (3.07–5.01) 4.65 (3.54–5.76)	17.0 (7.6–26.4) 18.4 (10.8–26.0)	NI	N₂O, O₂, and halothane or IV thiamylal	Halothane; N₂O and O₂	NI
Hong, 1997	RCT	T&A	I	ETT	24/25	5.8 (4.30–15.95) 6.4 (4.80–8.00)	20.8 (17.09–24.52) 22.4 (18.96–25.85)	36 (72)	Enflurane	Enflurane, O_2_ and air	NI
von Ungern-Sternberg, 2013	RCT	T&A	I‒III	ETT	50/50	5 (1–14) 4 (1–14)	20 (12.7–84.9) 18 (12.9–64.0)	41 (41)	Sevoflurane or IV propofol	Sevoflurane and N₂O	34/36
Baijal, 2015	Observational	T&A	NI	ETT	303/577	5.0 (1.00–17.00) 4.0 (1.00–19.00)	19.4 (6.00–157.00) 18.56 (6.7–99.5)	NI	Sevoflurane	Sevoflurane	225/94
Ramgolam, 2018	RCT	T w/wo A	I‒II	LMA	140/143	6.8 (1.0–16.3) 6.4 (1.1–16.0)	25.3 (11.0–84.6) 23.4 (9.9–99.0)	133 (47)	Sevoflurane or IV propofol	Sevoflurane	76/87
Kapoor, 2025	RCT	T w/wo A	I‒II	LMA	62/62	4.54 (1.33–7.00) 4.63 (2.00–6.75)	19.60 (3.00–36.70) 18.65 (11.30–36.30)	72 (58)	Sevoflurane w/wo N₂O	Sevoflurane	NI
Wan, 2026	RCT	T&A	I‒II	ETT	50/47	5.18 ± 1.61 5.13 ± 1.53	23.2 ± 9.29 21.1 ± 5.76	63 (65)	Sevoflurane with IV propofol	Sevoflurane	50/47

A, Adenoidectomy; ASA-PS, American Society of Anesthesiologists Physical Status Classification; ETT, Endotracheal Tube; IV, Intravenous; LMA, Laryngeal Mask; NI, Not Informed; OSA: Obstructive Sleep Apnea; RCT, Randomized Controlled Trial; SD, Standard Deviation; T&A, Adenotonsillectomy; T/A, Tonsillectomy and/or Adenoidectomy; T, Tonsillectomy; w/wo, with/without.

a Mean ± SD or Median (range).

**Figure 1:**
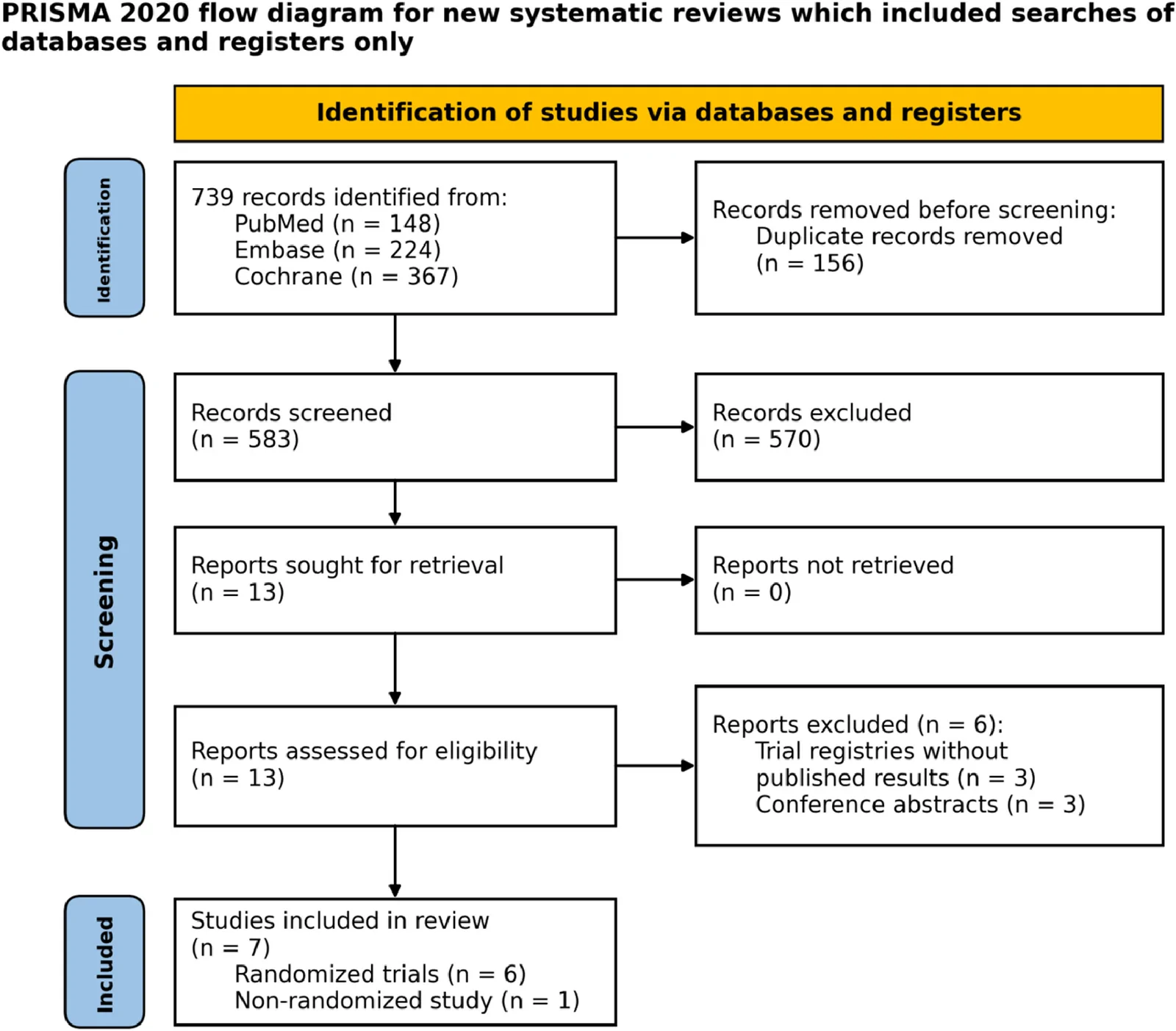
PRISMA 2020 flowchart.

### Primary analysis: randomized controlled trials

No significant differences were identified between groups in the incidence of laryngospasm (RR = 0.99; 95% CI 0.43–2.30; p = 0.984; I² = 0%; Fig. 2A). The incidence of cough (RR = 0.57; 95% CI 0.43–0.75; p < 0.001; I² = 28.3%; Fig. 2C) and desaturation (RR = 0.62; 95% CI 0.44–0.88; p = 0.007; I² = 32.6%; Fig. 2B) were significantly lower in the deep extubation group compared with the awake group. When stratified by airway device, no significant difference was observed among patients extubated with an ETT (RR = 0.54; 95% CI 0.27–1.06; p = 0.072; I² = 44.6%; Fig. 2C), whereas the reduction in cough incidence remained statistically significant in the LMA subgroup (RR = 0.53; 95% CI 0.30–0.96; p = 0.035; I² = 35.3%; Fig. 2C), and the test for subgroup differences provided no evidence of effect modification by airway device (p = 0.992). In contrast, airway obstruction occurred more frequently following deep extubation (RR = 1.82; 95% CI 1.10–3.03; p = 0.020; I² = 0%; Fig. 2D).

**Figure 2:**
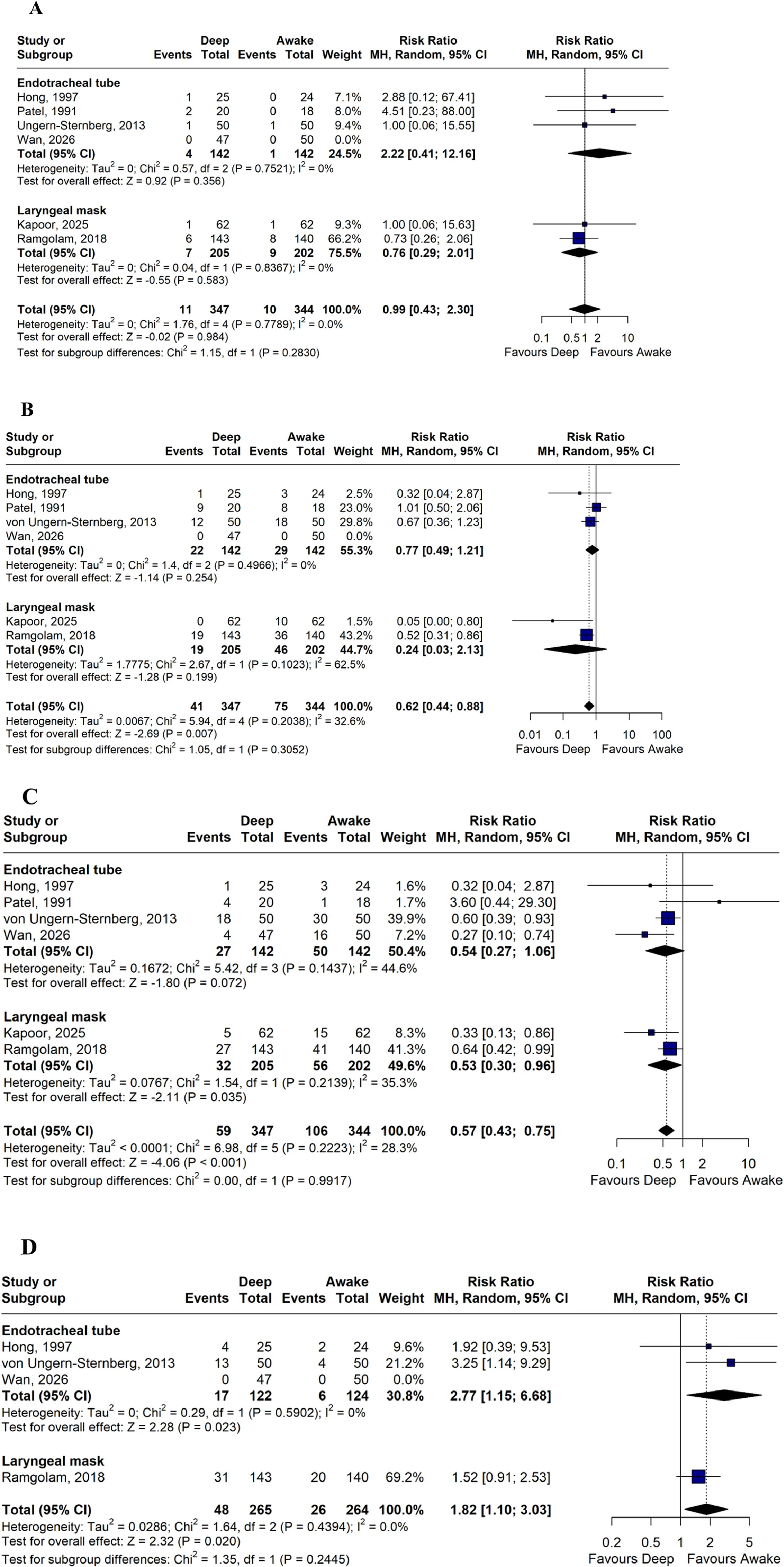
**Respiratory complications in RCTs.** (A) Incidence of laryngospasm. (B) Incidence of desaturation. (C) Incidence of cough. (D) Incidence of airway obstruction.

Subgroup analyses by type of airway device yielded similar findings: among studies using an ETT, neither laryngospasm (RR = 2.22; 95% CI 0.41–12.16; p = 0.356; I² = 0%; Fig. 2A) nor desaturation (RR = 0.77; 95% CI 0.49–1.21; p = 0.254; I² = 0%; Fig. 2B) differed significantly between deep and awake extubation.

### Sensitivity analysis including the observational study

When the observational study by Baijal et al. was included in the sensitivity analysis, the pooled estimate for desaturation was attenuated and became non-significant (RR = 0.72; 95% CI 0.51–1.02; p = 0.063; I² = 44.5%; Fig. 3), suggesting that study design may have influenced this outcome. Laryngospasm, cough, or airway obstruction were not influenced by Baijal et al.

**Figure 3:**
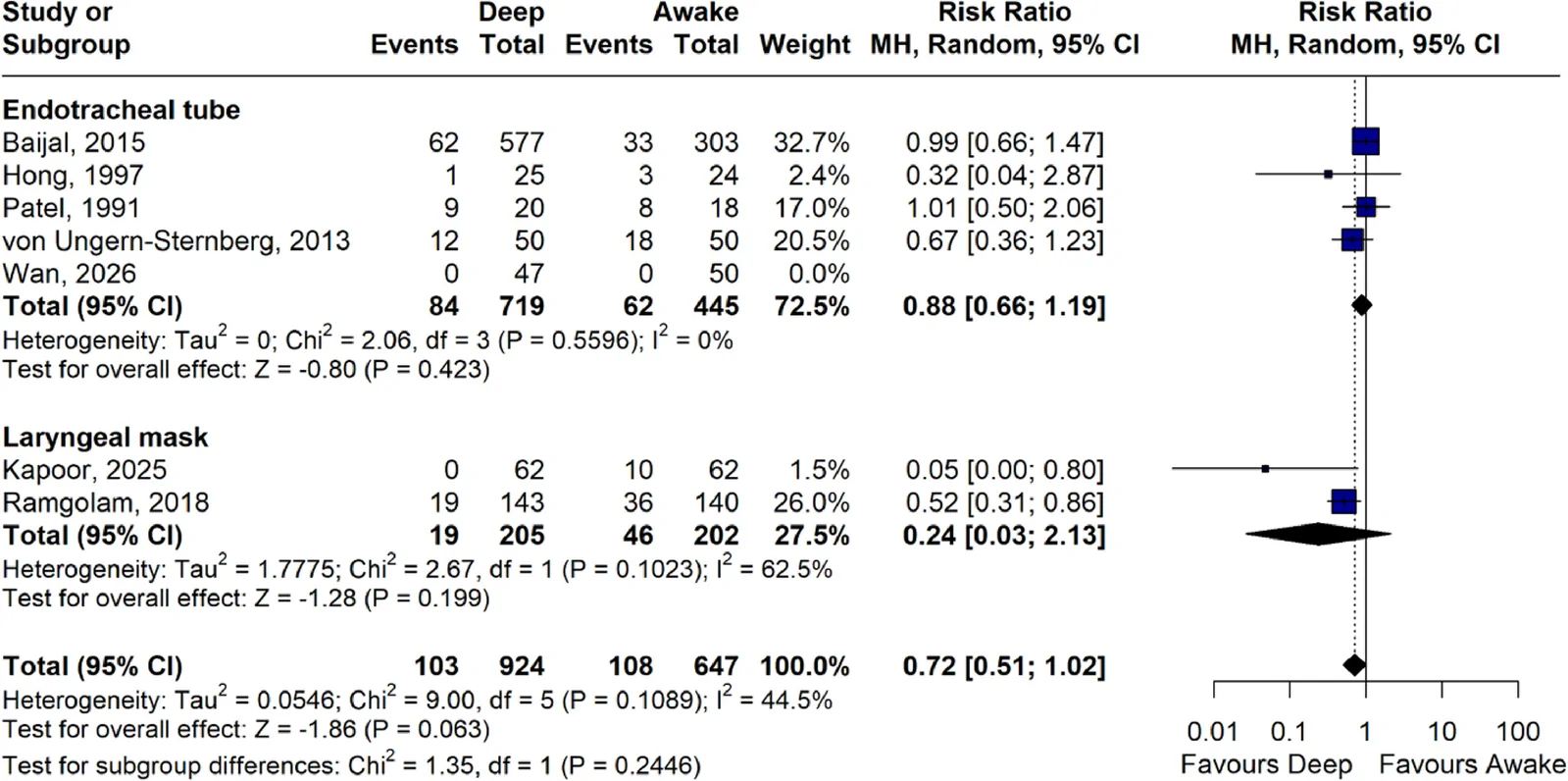
**Sensitivity analysis including the observational study.** Incidence of desaturation.

### Exploratory subgroup analysis with sevoflurane-based maintenance anesthesia

In the subgroup analysis of patients with sevoflurane-based maintenance anesthesia,^10^^,^^11^^,^^21^^,^^24^^,^^25^ the risk of laryngospasm showed no significant difference between the awake and deep extubation groups (RR = 0.79; 95% CI 0.32–1.96; p = 0.60; I² = 0%; Fig. 4A). Regarding airway obstruction, the results showed no significant difference between the awake and deep group (RR = 1.92; 95% CI 0.96–3.84; p = 0.06; I² = 38.8%; Fig. 4D). In contrast, the incidence of cough was significantly higher in the awake group (RR = 0.55; 95% CI 0.42–0.73; p < 0.001; I² = 19%; Fig. 4C), while for desaturation, the only outcome with the observational study, rates showed no statistically significant difference between the two techniques (RR = 0.67; 95% CI 0.44–1.04; p = 0.07; I² = 61%; Fig. 4B).

**Figure 4:**
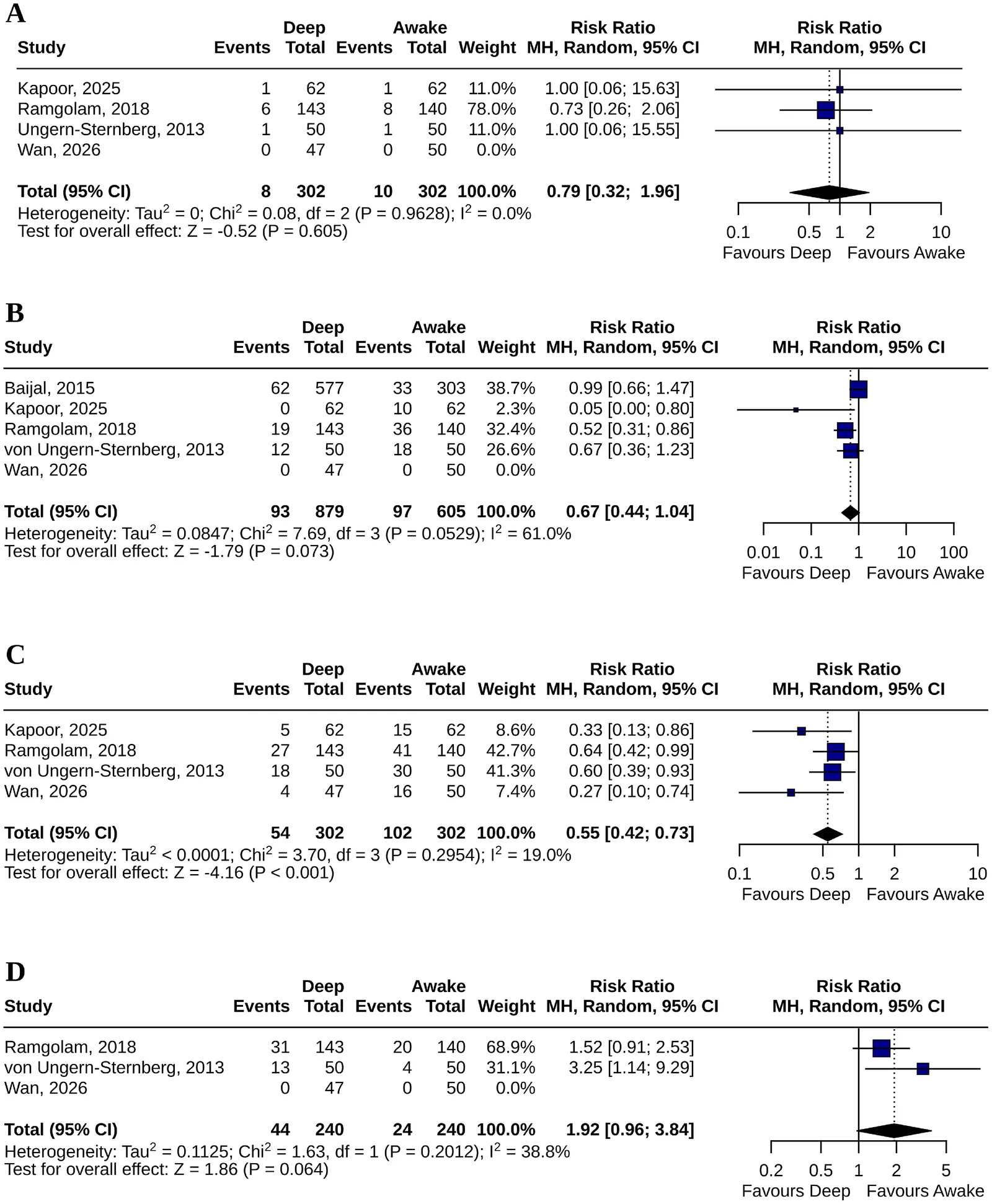
**Exploratory subgroup analysis with sevoflurane-based maintenance anesthesia.** (A) Incidence of laryngospasm. In the subgroup restricted to studies using sevoflurane for maintenance, deep airway device removal was not associated with a significantly different risk of laryngospasm compared with awake removal (RR = 0.79; 95% CI 0.32–1.96; p = 0.605; I² = 0%). (B) Incidence of desaturation. In the subgroup restricted to studies using sevoflurane-based maintenance anesthesia, deep airway device removal was not associated with a significantly different risk of oxygen desaturation compared with awake removal (RR = 0.67; 95% CI 0.44–1.04; p = 0.073; I² = 61.0%). (C) Incidence of cough. In the subgroup restricted to studies using sevoflurane-based maintenance anesthesia, deep airway device removal was associated with a significantly lower risk of cough compared with awake removal (RR = 0.55; 95% CI 0.42–0.73; p < 0.001; I² = 19%). (D) Incidence of airway obstruction. In the subgroup restricted to studies using sevoflurane-based maintenance anesthesia, deep airway device removal was not associated with a significantly different risk of airway obstruction compared with awake removal (RR = 1.92; 95% CI 0.96–3.84; p = 0.064; I² = 38.8%). CI, Confidence Interval; df, Degrees of freedom; I², Inconsistency statistic; MH, Mantel-Haenszel; RR, Risk Ratio; τ², Between-study variance.

### Leave-one-out analysis

Sensitivity analyses using the leave-one-out approach demonstrated that the overall findings were generally robust for most outcomes, with a notable exception for airway obstruction. The leave-one-out analysis for laryngospasm yielded no significant new findings (Suppl. Fig. 1). In the analysis of desaturation, results were consistent with the main findings, except when Baijal et al. was excluded, which rendered the difference statistically significant in favor of deep extubation; both Baijal et al. and Ramgolam et al. were flagged as outliers for this outcome (Suppl. Fig. 2). For cough, statistical significance persisted regardless of the study omitted, although Patel et al. was identified as a potential outlier (Suppl. Fig. 3). For airway obstruction, the pooled effect lost significance when either Hong et al. or von Ungern-Sternberg et al. was omitted, indicating that the significant pooled effect for this outcome is driven by these two studies and should be interpreted with caution (Suppl. Fig. 4).

### Risk of Bias

The assessment of publication bias in this review was constrained by the small number of eligible studies (n = 7). Because statistical methods such as funnel plot inspection and Egger’s regression are unreliable with fewer than 10 studies, a formal quantitative evaluation was not conducted. Instead, we relied on a qualitative appraisal of risk across the included studies.

Among the RCTs, three raised “some concerns” regarding the risk of bias; these were primarily related to the randomization process (Hong, 1997), deviations from intended intervention (Wan, 2026), selection of the reported result (Wan, 2026) and measurement of the outcome (Kapoor, 2025; Wan, 2026). The remaining three RCTs (Ramgolam, 2018; von Ungern-Sternberg, 2013; and Patel, 1991) were considered to have a low risk of bias. The observational study (Baijal, 2015) was judged to carry a higher risk, particularly due to potential confounding and issues in the classification of interventions, since the choice between awake and deep extubation was left entirely to the attending anesthesiologist without standardized criteria, leading to possible misclassification. In addition, outcome measurement relied on voluntary self-reporting by anesthesiologists and PACU nurses, which may have introduced detection bias and underreporting of respiratory complications.

### Certainty of evidence

Using the GRADE approach, the overall certainty of evidence was rated as high for cough. For laryngospasm and airway obstruction, certainty was downgraded to moderate due to serious imprecision: the confidence interval for laryngospasm was wide, crossing the line of no effect with few events across studies; for airway obstruction, only four trials with 529 participants contributed to this outcome, and the pooled estimate was sensitive to the exclusion of individual studies in leave-one-out analyses. For desaturation assessed in the RCT-only primary analysis, certainty was also downgraded to moderate due to serious inconsistency, as heterogeneity was observed (I² = 32.6%) and the pooled estimate was sensitive to study design and to individual studies in leave-one-out analyses. For desaturation assessed in the non-randomized study, certainty was rated as very low, as non-randomized evidence starts at low certainty by GRADE default and was further downgraded for serious risk of bias related to confounding, classification of the intervention, and outcome measurement (Suppl. Table 3).

## Discussion

We conducted a systematic review and meta-analysis of six RCTs and one observational study, including 1,571 pediatric patients undergoing tonsillectomy or adenotonsillectomy, to compare awake versus deep removal of airway devices on adverse respiratory outcomes. To reduce confounding and intervention-classification bias, the primary meta-analysis was restricted to randomized controlled trials, while the observational study was incorporated as sensitivity analysis when outcome data were available.

Deep airway device removal was associated with a higher incidence of airway obstruction and no difference in laryngospasm. Although desaturation was lower with deep removal in the RCT-only analysis, this finding was attenuated after inclusion of the observational study and should be interpreted cautiously. The overall pooled analysis suggested a significant reduction in cough consistent with earlier trials.^8^, ^9^, ^10^^,^^21^^,^^23^^,^^26^ Although this reduction reached statistical significance only in the LMA subgroup, the point estimates were nearly identical in the two subgroups and the test for subgroup differences did not suggest effect modification. Statistical significance in one subgroup and its absence in another does not, by itself, establish a differential effect; the wide confidence interval in the ETT subgroup most likely reflects imprecision. Although the absence of LMA contact with the vocal cords and trachea offers a plausible mechanism, thereby avoiding direct stimulation of perilaryngeal structures capable of provoking coughing. This is particularly relevant in otolaryngologic procedures, where coughing may lead to complications such as suture dehiscence, bleeding, or airway trauma.

Deep airway device removal is more frequently associated with upper airway obstruction than awake airway device removal.^25^ This association was also observed in our population of adenotonsillectomy and tonsillectomy patients. Because pharyngeal muscles recover more slowly from anesthesia than the diaphragm and intercostals, the upper airway is prone to collapse after extubation. When pharyngeal tone cannot counter negative inspiratory pressure, obstruction may occur; therefore, awake extubation is preferred in patients susceptible to residual anesthetic effects.^27^ To prevent or manage airway obstruction, supportive measures include placing shoulder rolls, positioning the patient laterally to reduce the impact on oral structures (soft palate, tongue, and tonsils), or turning the head to the side. In neonates and infants, moderate CPAP delivered via mask is particularly effective in maintaining airway patency and preventing collapse.^27^

Among airway complications, laryngospasm and desaturation pose life-threatening risks,^28^ accounting for 27% of perioperative cardiac arrests in children, with 6% directly attributable to laryngospasm.^29^ In our analysis, no significant difference was observed among groups regarding the incidence of laryngospasm. Baijal et al.^25^ attributed neutral findings to the presence of subspecialty-trained pediatric anesthesiologists and dedicated PACU nurses, whereas Ramgolam et al. suggested that one-to-one nursing ratios enabled immediate intervention and prevented escalation.^11^

Although the RCT-only analysis favored deep airway device removal, this benefit lost significance in the combined analysis with the observational study and with the sevoflurane subgroup due to residual confounding and clinical heterogeneity. Desaturation is a complex endpoint influenced by variable threshold definitions, perioperative oxygen supplementation, rescue maneuvers, staff expertise, anesthetic era, and study design. Critically, none of the included studies were designed to systematically capture post-removal rescue interventions, rendering causal inferences speculative. Given this interplay of confounders, desaturation findings should not be interpreted as definitive evidence favoring either extubation strategy.

Interpretation must account for the anesthetic context of the underlying evidence. Much of the available literature originates from earlier anesthetic eras, including the use of agents no longer employed in contemporary pediatric practice.^30^ Desaturation thresholds varied widely, often defined as SpO_2_ values between 92% and 95% sustained for short durations, levels that may not reflect clinically meaningful hypoxemia in otherwise healthy children. Moreover, oxygen desaturation is not an independent adverse event but rather a downstream physiological consequence of other airway phenomena, such as laryngospasm, upper airway obstruction, coughing, or transient disruption of oxygen delivery during emergence.^31^

Notably, results were consistent in the sevoflurane subgroup. This agent’s low blood-gas solubility causes rapid emergence, often associated with increased airway irritability and emergence delirium in children.^32^ Specifically, our findings reinforce that while the awake technique shows a strong trend toward reducing airway obstruction (p = 0.064), it significantly increases the incidence of coughing, regardless of sevoflurane's known irritant potential during emergence.^10^^,^^32^ In the overall pooled analysis for airway obstruction, the significant effect was primarily driven by the studies of Hong et al. and von Ungern-Sternberg et al. These studies utilized endotracheal tubes and involved older anesthetic agents (enflurane and sevoflurane/nitrous oxide, respectively), which likely amplified the risk of upper airway collapse during deep extubation compared to the more homogeneous sevoflurane subgroup. These findings across a more homogeneous subgroup strengthen the evidence that the choice between awake and deep extubation should be tailored to the patient's specific risk for obstruction versus irritability.

Clinical heterogeneity was also introduced by variation in anesthetic maintenance. Five studies used sevoflurane, while one used halothane and another enflurane, agents with substantially different pharmacokinetic profiles and airway irritability compared with contemporary practice. As anesthetic maintenance directly influences emergence physiology and upper airway reactivity, pooling across anesthetic eras may introduce heterogeneity beyond what statistical methods can fully capture, reinforcing the exploratory nature of the overall pooled analysis.

LMA removal and ETT extubation were pooled because the intervention under investigation was the depth of anesthesia at the time of device removal, not the type of device itself. Nevertheless, subgroup analyses by device type were performed to explore differential effects, and clinically meaningful differences were observed for cough and airway obstruction, reinforcing the importance of interpreting results at the subgroup level. Although this reduction reached statistical significance only in the LMA subgroup, the point estimates were nearly identical in the two subgroups and the test for subgroup differences did not suggest effect modification (p = 0.992).

The pharmacological profile of sevoflurane further underscores the clinical importance of this decision, particularly in high-risk subgroups. Patients with OSA or active respiratory symptoms frequently exhibit airway hyperreactivity; when combined with sevoflurane anesthesia, this state may exacerbate inflammatory responses and consequently elevate the incidence of PRAEs.^33^ Children with OSA represent an important high-risk population in whom the balance between airway obstruction and airway irritability deserves particular consideration.

Awake extubation is generally recommended in children with difficult airways, OSA, prematurity, or an increased risk of regurgitation and aspiration.^27^ Conversely, deep extubation may be advantageous in patients with asthma, nocturnal cough, or recent upper respiratory infections, where minimizing airway reflexes is desirable.^27^ Regardless of the chosen approach, maintaining an adequate depth of anesthesia is the most critical factor for safe airway device removal. Extubation performed in an intermediate state carries the greatest risk, as it predisposes patients to airway obstruction and other serious complications.^27^

A clinically important consideration is that the outcomes assessed differ substantially in severity. Cough, while undesirable in otolaryngologic surgery due to concerns about wound disruption or bleeding, is generally self-limited and rarely threatens patient safety.^33^^,^^34^ Airway obstruction, laryngospasm and clinically significant desaturation represent true airway emergencies. Therefore, the respiratory outcomes in this meta-analysis cannot be treated as clinically equivalent. While deep extubation reduces the incidence of a minor reflex (cough), its association with an increased risk of a severe complication (airway obstruction) represents a shift in the risk profile from minor physiological disruption to potential patient harm. This hierarchy of severity must be weighed when choosing the extubation timing, as avoiding a minor reflex does not justify increasing the risk of a life-threatening event.

### Limitations

This review has several limitations. First, several included studies were conducted during earlier anesthetic eras, incorporating agents no longer used in contemporary pediatric anesthesia, which limits external validity. Second, the inclusion of one observational study alongside randomized controlled trials may have introduced confounding. Third, the relatively small number of eligible studies precluded a formal assessment of publication bias. Fourth, most studies were conducted in tertiary centers with highly trained pediatric anesthesiologists and recovery staff, which may limit the generalizability of our findings to other settings. Fifth, one trial administered dexmedetomidine only in the deep extubation arm, which may have contributed to the reduction in cough observed in that study.^24^

Taken together, these considerations demonstrate that the observed differences between awake and deep airway device removal in this review should be interpreted as manifestations of well-described physiological trade-offs inherent to anesthetic depth and airway reflex recovery. They do not constitute definitive evidence to guide contemporary clinical practice, particularly in the current sevoflurane and TIVA dominant era.^33^ Future studies should prioritize standardized, clinically meaningful outcome definitions and explicitly account for modern anesthetic maintenance strategies to generate practice-relevant evidence.

## Conclusion

In pediatric patients undergoing tonsillectomy and adenotonsillectomy, deep airway device removal was associated with less coughing, with no evidence that this effect differed between LMA and ETT. Deep airway device removal was also associated with a higher incidence of airway obstruction compared with awake removal. No significant differences were observed in laryngospasm. Desaturation findings were influenced by study design and became non-significant after inclusion of observational evidence; therefore, they should not be interpreted as conclusive evidence favoring either strategy. Future clinical trials should be adequately powered, restricted to clearly defined surgical populations, incorporate standardized and clinically meaningful outcome measures and explicitly account for contemporary anesthetic maintenance strategies.

## Data availability statement

All data supporting the findings of this study are available within the article itself and its Supplementary Material. The study-level dataset extracted for this meta-analysis, as well as the statistical code and analysis scripts used, are available from the corresponding author upon reasonable request.

## Declaration of generative AI and AI-assisted technologies in the manuscript preparation process

During the preparation of this work, the author(s) used ChatGPT (OpenAI) in order to review and improve the language and readability of the manuscript. After using this tool/service, the authors reviewed and edited the content as needed and take full responsibility for the content of the published article.

## Authors’ contributions

Henrique B. Pivetta: Conceptualization; methodology; literature search; study selection; statistical analysis; writing; editing; risk of bias assessment. Alice O. Almeida: Study selection; data extraction; writing. Rafael L. Miranda: Statistical analysis; writing. Rafaela P. Silva: Writing; data extraction. Raphaela G. Mendes: Writing; data extraction; review; study selection. João M.M. Borges: Writing; data extraction. Raquel E. Saldanha: Writing; data extraction. Matheus Arraes: Risk of bias assessment; review. Pablo P. Sanín: Supervision; Conceptualization; writing; review. Walid Alrayashi: Supervision; Conceptualization; writing; review.

## Funding

This research did not receive any specific grant from funding agencies in the public, commercial, or non-profit sectors.

## Conflicts of interest

The authors declare no conflicts of interest.

## Appendix Supplementary materials

Image, application 1

## Footnotes

Supplementary material associated with this article can be found in the online version at doi:10.1016/j.bjane.2026.844840.
