# Supplementary material for: Awake versus deep airway device removal in pediatric tonsillectomy: a systematic review and meta-analysis
Source: Braz J Anesthesiol. 2026 Oct 9;76(6):844840. doi: 10.1016/j.bjane.2026.844840 (PMC13742090; doi:10.1016/j.bjane.2026.844840)
Supplement: Supplementary file 1 [file mmc1.docx]

BJAN-D-26-00232_Supplementary Material

**Supplementary Methods 1** PRISMA checklist.

| **Section and Topic** | **Item #** | **Checklist item** |
| --- | --- | --- |
| **TITLE** | | |
| Title | 1 | Identify the report as a systematic review. |
| **ABSTRACT** | | |
| Abstract | 2 | See the PRISMA 2020 for Abstracts checklist. |
| **INTRODUCTION** | | |
| Rationale | 3 | Describe the rationale for the review in the context of existing knowledge. |
| Objectives | 4 | Provide an explicit statement of the objective(s) or question(s) the review addresses. |
| **METHODS** | | |
| Eligibility criteria | 5 | Specify the inclusion and exclusion criteria for the review and how studies were grouped for the syntheses. |
| Information sources | 6 | Specify all databases, registers, websites, organizations, reference lists and other sources searched or consulted to identify studies. Specify the date when each source was last searched or consulted. |
| Search strategy | 7 | Present the full search strategies for all databases, registers and websites, including any filters and limits used. |
| Selection process | 8 | Specify the methods used to decide whether a study met the inclusion criteria of the review, including how many reviewers screened each record and each report retrieved, whether they worked independently, and if applicable, details of automation tools used in the process. |
| Data collection process | 9 | Specify the methods used to collect data from reports, including how many reviewers collected data from each report, whether they worked independently, any processes for obtaining or confirming data from study investigators, and if applicable, details of automation tools used in the process. |
| Data items | 10a | List and define all outcomes for which data were sought. Specify whether all results that were compatible with each outcome domain in each study were sought (e.g., for all measures, time points, analyses), and if not, the methods used to decide which results to collect. |
|  | 10b | List and define all other variables for which data were sought (e.g., participant and intervention characteristics, funding sources). Describe any assumptions made about any missing or unclear information. |
| Study risk of bias assessment | 11 | Specify the methods used to assess risk of bias in the included studies, including details of the tool(s) used, how many reviewers assessed each study and whether they worked independently, and if applicable, details of automation tools used in the process. |
| Effect measures | 12 | Specify for each outcome the effect measure(s) (e.g., risk ratio, mean difference) used in the synthesis or presentation of results. |
| Synthesis methods | 13a | Describe the processes used to decide which studies were eligible for each synthesis (e.g., tabulating the study intervention characteristics and comparing against the planned groups for each synthesis (item #5)). |
|  | 13b | Describe any methods required to prepare the data for presentation or synthesis, such as handling of missing summary statistics, or data conversions. |
|  | 13c | Describe any methods used to tabulate or visually display results of individual studies and syntheses. |
|  | 13d | Describe any methods used to synthesize results and provide a rationale for the choice(s). If meta-analysis was performed, describe the model(s), method(s) to identify the presence and extent of statistical heterogeneity, and software package(s) used. |
|  | 13e | Describe any methods used to explore possible causes of heterogeneity among study results (e.g., subgroup analysis, meta-regression). |
|  | 13f | Describe any sensitivity analyses conducted to assess robustness of the synthesized results. |
| Reporting bias assessment | 14 | Describe any methods used to assess risk of bias due to missing results in a synthesis (arising from reporting biases). |
| Certainty assessment | 15 | Describe any methods used to assess certainty (or confidence) in the body of evidence for an outcome. |
| **RESULTS** | | |
| Study selection | 16a | Describe the results of the search and selection process, from the number of records identified in the search to the number of studies included in the review, ideally using a flow diagram. |
|  | 16b | Cite studies that might appear to meet the inclusion criteria, but which were excluded, and explain why they were excluded. |
| Study characteristics | 17 | Cite each included study and present its characteristics. |
| Risk of bias in studies | 18 | Present assessments of risk of bias for each included study. |
| Results of individual studies | 19 | For all outcomes, present, for each study: (a) summary statistics for each group (where appropriate) and (b) an effect estimates and its precision (e.g., confidence/credible interval), ideally using structured tables or plots. |
| Results of syntheses | 20a | For each synthesis, briefly summarize the characteristics and risk of bias among contributing studies. |
|  | 20b | Present results of all statistical syntheses conducted. If meta-analysis was done, present for each the summary estimate and its precision (e.g., confidence/credible interval) and measures of statistical heterogeneity. If comparing groups, describe the direction of the effect. |
|  | 20c | Present results of all investigations of possible causes of heterogeneity among study results. |
|  | 20d | Present results of all sensitivity analyses conducted to assess the robustness of the synthesized results. |
| Reporting biases | 21 | Present assessments of risk of bias due to missing results (arising from reporting biases) for each synthesis assessed. |
| Certainty of evidence | 22 | Present assessments of certainty (or confidence) in the body of evidence for each outcome assessed. |
| **DISCUSSION** | | |
| Discussion | 23a | Provide a general interpretation of the results in the context of other evidence. |
|  | 23b | Discuss any limitations of the evidence included in the review. |
|  | 23c | Discuss any limitations of the review processes used. |
|  | 23d | Discuss implications of the results for practice, policy, and future research. |
| **OTHER INFORMATION** | | |
| Registration and protocol | 24a | Provide registration information for the review, including register name and registration number, or state that the review was not registered. |
|  | 24b | Indicate where the review protocol can be accessed, or state that a protocol was not prepared. |
|  | 24c | Describe and explain any amendments to information provided at registration or in the protocol. |
| Support | 25 | Describe sources of financial or non-financial support for the review, and the role of the funders or sponsors in the review. |
| Competing interests | 26 | Declare any competing interests of review authors. |
| Availability of data, code and other materials | 27 | Report which of the following are publicly available and where they can be found: template data collection forms; data extracted from included studies; data used for all analyses; analytic code; any other materials used in the review. |

**Supplementary Methods 2** Full search strategy.

**PubMed**

(children OR paediatric OR infants OR child OR toddler) AND (tonsillectomy OR adenotonsillectomy) AND (timing OR awake OR wakeful OR conscious OR asleep OR deep OR deeply OR depth OR unconscious) AND (endotracheal OR extubation OR ETT OR "Intubation, Intratracheal"[Mesh] OR LMA OR "Laryngeal Masks"[Mesh] OR airway)

**Embase**

('children' OR 'paediatric' OR 'infants' OR 'child' OR 'toddler') AND ('tonsillectomy' OR 'adenotonsillectomy') AND ('timing' OR 'awake' OR 'wakeful' OR 'conscious' OR 'asleep' OR 'deep' OR 'deeply' OR 'depth' OR 'unconscious') AND ('endotracheal' OR 'extubation' OR 'ett' OR 'endotracheal intubation'/exp OR 'lma' OR 'laryngeal mask'/exp OR 'airway')

**Cochrane**

(children OR paediatric OR infants OR child OR toddler) AND (tonsillectomy OR adenotonsillectomy) AND (timing OR awake OR wakeful OR conscious OR asleep OR deep OR deeply OR depth OR unconscious) AND (endotracheal OR extubation OR ETT OR "Intubation, Intratracheal" OR LMA OR "Laryngeal Masks" OR airway)

**Supplementary Table 1** Risk of bias summary for randomized studies (RoB2).

| **Study** | **Bias from randomization process** | **Bias due to deviations from intended interventions** | **Bias due to missing outcome data** | **Bias in measurement of the outcomes** | **Bias in selection of the reported result** | **Overall risk of bias** |
| --- | --- | --- | --- | --- | --- | --- |
| Hong 1997 | Some concerns | Low | Low | Low | Low | Some concerns |
| Kapoor 2025 | Low | Low | Low | Some concerns | Low | Some concerns |
| Patel 1991 | Low | Low | Low | Low | Low | Low |
| Ramgolam 2018 | Low | Low | Low | Low | Low | Low |
| Von Ungern-Sternberg 2013 | Low | Low | Low | Low | Low | Low |
| Wan 2026 | Low | Some concerns | Low | Some concerns | Some concerns | Some concerns |

**Supplementary Table 2** Risk of Bias in Non-randomized Studies of Interventions (ROBINS-I).

**Supplementary Table 3** GRADE assessment.

**Supplementary Figure 1** Leave-one-out analysis forest plot for laryngospasm. Forest plot displaying the leave-one-out sensitivity analysis for laryngospasm, in which each study was sequentially omitted to assess its individual influence on the pooled estimate. The overall pooled risk ratio (RR = 0.99; 95% CI 0.43‒2.30; p = 0.984; I² = 0%) remained stable and non-significant regardless of the study omitted, indicating robustness of the main finding. CI, Confidence Interval; I², Inconsistency Statistic; RR, Risk Ratio.

**Supplementary Figure 2** Leave-one-out analysis forest plot for desaturation. Forest plot displaying the leave-one-out sensitivity analysis for oxygen desaturation, in which each study was sequentially omitted to assess its individual influence on the pooled estimate. The overall pooled risk ratio was non-significant (RR = 0.72; 95% CI 0.51‒1.02; p = 0.063; I² = 44.5%). When Baijal et al. was omitted, the pooled estimate became statistically significant in favor of deep removal (RR = 0.64; 95% CI 0.45‒0.91; p = 0.014), identifying this study as an outlier. Ramgolam et al. was also flagged as an outlier for this outcome. CI, Confidence Interval; I², Inconsistency Statistic; RR, Risk Ratio.

**Supplementary Figure 3** Leave-one-out analysis forest plot for cough. Forest plot displaying the leave-one-out sensitivity analysis for cough, in which each study was sequentially omitted to assess its individual influence on the pooled estimate. The overall pooled risk ratio remained statistically significant in favor of deep removal (RR = 0.57; 95% CI 0.43–0.75; p < 0.001; I² = 28.3%) regardless of the study omitted, indicating robustness of the main finding. Patel et al. was identified as a potential outlier, as its omission yielded the largest reduction in heterogeneity (I² = 0%). CI, Confidence Interval; I², Inconsistency statistic; RR, Risk Ratio.

**Supplementary Figure 4** Leave-one-out analysis forest plot for airway obstruction. Forest plot displaying the leave-one-out sensitivity analysis for airway obstruction, in which each study was sequentially omitted to assess its individual influence on the pooled estimate. The overall pooled risk ratio favored awake removal (RR = 1.82; 95% CI 1.10‒3.03; p = 0.020; I² = 0%). Statistical significance was lost when either Hong et al. (RR = 1.92; 95% CI 0.96‒3.84; p = 0.064) or von Ungern-Sternberg et al. (RR = 1.55; 95% CI 0.95‒2.52; p = 0.078) was omitted, indicating that the pooled estimate for this outcome is sensitive to these two studies. CI, Confidence Interval; I², Inconsistency Statistic; RR, Risk Ratio.
